# Epidemics in Interconnected Small-World Networks

**DOI:** 10.1371/journal.pone.0120701

**Published:** 2015-03-23

**Authors:** Meng Liu, Daqing Li, Pengju Qin, Chaoran Liu, Huijuan Wang, Feilong Wang

**Affiliations:** 1 School of Reliability and Systems Engineering, Beihang University, Beijing, China; 2 Science and Technology on Reliability and Environmental Engineering Laboratory, Beijing, China; 3 Faculty of Electrical Engineering, Mathematics, and Computer Science, Delft University of Technology, Delft, The Netherlands; Universidad Rey Juan Carlos, SPAIN

## Abstract

Networks can be used to describe the interconnections among individuals, which play an important role in the spread of disease. Although the small-world effect has been found to have a significant impact on epidemics in single networks, the small-world effect on epidemics in interconnected networks has rarely been considered. Here, we study the susceptible-infected-susceptible (SIS) model of epidemic spreading in a system comprising two interconnected small-world networks. We find that the epidemic threshold in such networks decreases when the rewiring probability of the component small-world networks increases. When the infection rate is low, the rewiring probability affects the global steady-state infection density, whereas when the infection rate is high, the infection density is insensitive to the rewiring probability. Moreover, epidemics in interconnected small-world networks are found to spread at different velocities that depend on the rewiring probability.

## Introduction

Epidemic dynamics in complex networks [1–3] have been extensively studied, with respect to the epidemic threshold and the infection density over time, in single networks [4–7], such as Erdős–Rényi (ER) networks [8], Watts and Strogatz (WS) networks [9] and scale-free (SF) networks [10]. However, epidemics can also spread across multiple communities or species [11],which are not limited to a single network. For example, a disease may spread from the animal interaction network to the human interaction network via human-animal interactions.

Epidemic spreading in interconnected networks has been recently studied. Most of these studies investigated the susceptible-infected-susceptible (SIS) [12–14] or the susceptible-infected-recovered (SIR) [15–18] epidemic spreading model. It has been demonstrated [12] that the epidemic threshold in two interconnected random networks can be lower than that in each isolated network. In [15], Dickison et al. investigated epidemic spreading in interconnected networks and found two different regimes: in the strongly coupled case, the disease either spreads in both networks or does not spread at all; in the weakly coupled case, there exists an intermediate scenario in which the epidemic spreads in only one network. Buono et al. [17] reported a study of the SIR process in partially overlapped multiplex networks and developed a theoretical framework to determine the effect of the overlap fraction on the spread of a disease. Li et al. in [14] have illustrated how the spatial constraint [19,20] of the interconnection links between lattices affects the epidemic threshold and the infection density.

Many real-world networks are neither regular networks nor random networks. The small-world model [9] proposed by Watts and Strogatz captures the features of high clustering and small average path length, which have been widely observed in real-world networks. These small-world features [21,22] have been found to have a significant impact on the dynamics in interconnected networks [23,24]. The small-world model can be constructed from a regular lattice. In such a network, each existing link is randomly rewired with a rewiring probability *p*, which tunes the nature of the network between that of a regular network (*p* = 0) and that of a random network (*p* = 1).

**Fig 1:**
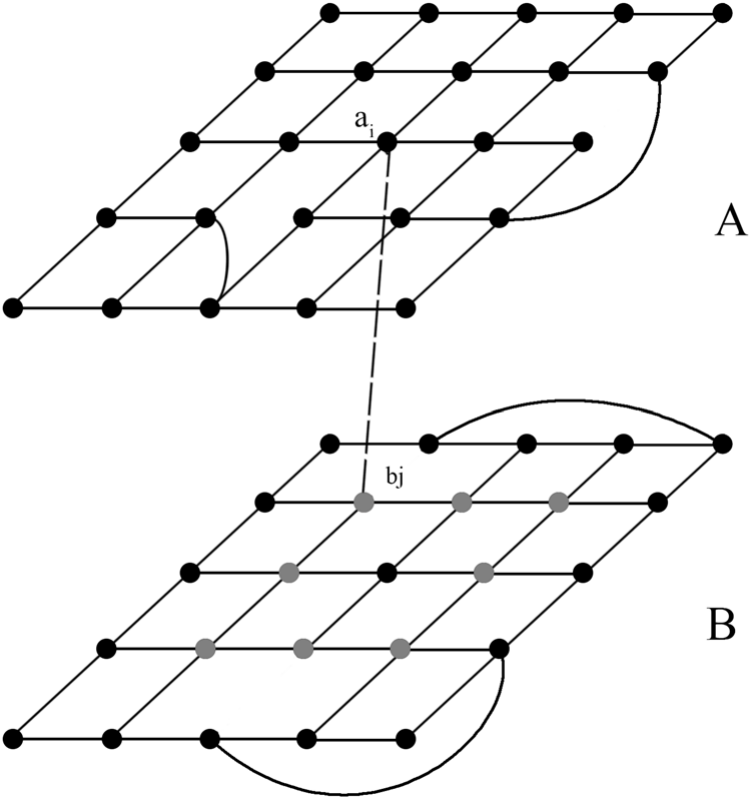
Interconnected small-world networks. The rewiring probability is *p* = 0.1. One randomly chosen node *a*_*i*_ in network A is connected to a node *b*_*j*_ in network B that is located at a spatial distance *R* from node *a*_*i*_, as defined in (Equation 1). When the spatial distance is specified as *R* = 1, for example, 8 nodes (gray) are at a spatial distance of 1 from node *a*_*i*_, and node *b*_*j*_ is randomly chosen among these 8 nodes.

In this paper, we analyze epidemic spreading in two interconnected small-world networks with the same rewiring probability (see Fig. 1). Because spatial constraints have been found to have a significant impact on many dynamical processes in networks [25–28], we account for such spatial constraints by requiring that the interconnection links can be only established from a node in one network to a node at a spatial distance *R* in the other network. We find that the epidemic threshold in such interconnected networks decreases when the rewiring probability of the small-world networks increases. When the infection rate is low, the rewiring probability affects the global steady-state infection density, whereas when the infection rate is high, the infection density is insensitive to the rewiring probability.

## Methods

### We generate interconnected networks as follows

1. A and B are two identical square lattices of linear size *L* and with *N* = L*L nodes. For each node *n*_*i*_ = *n*_0_, …*n*_*N-1*_ with lattice coordinates (*x*_*i*_,*y*_*i*_) and its neighbor *n*_j_ (*x*_*j*_,*y*_*j*_) in network A, remove the lattice link (*n*_*i*_,*n*_*j*_) that satisfies *x*_*i*_ < *x*_*j*_ or *y*_*i*_ < *y*_*j*_ and add a link between *n*_*i*_ and *n*_*k*_ with probability *p*_*A*_, where *n*_*k*_ is randomly chosen among all possible nodes, avoiding self-loops and duplicate links. The same process is also applied to network B with rewiring probability *p*_*B*_. In this paper, we consider *p*_*A*_ = *p*_*B*_ = *p*.

2. To construct an interconnection link, we first randomly choose a node *a_i_* in network A with lattice coordinates (*x*_*i*_,*y*_*i*_) and then randomly choose a node *b_j_* located at (*x*_*j*_,*y*_*j*_) in network B such that the following conditions are satisfied:

$$\begin{array}{l} \;\left|x_{i}-x_{j}\right|\leq R\;\text{and}\;\left|y_{i}-y_{j}\right|=R\;or\\ \left|x_{i}-x_{j}\right|=R\;\;\text{and}\;\left|y_{i}-y_{j}\right|\leq R. \end{array}$$ (1)

Then, we connect these two nodes with an interconnection link. We define the average coupling density as *q*; thus, there will be q*N interconnection links constructed between network A and network B. Each node may have multiple interconnection links, which differs from one to one interconnections. When *q* = 1, each node has one interconnection link on average, but not necessarily has exactly one interconnection link as in one to one interconnected network. It is possible that multiple interconnection links exist between the same pair of nodes.

In this paper, we consider the SIS epidemic spreading model. In this model, nodes can be in two possible states, susceptible (S) or infected (I). Initially, a fraction of randomly chosen nodes in network A are infected. At each time step, each susceptible node can be infected by each of its infected neighbors in the same network with probability *β*_1_ or by each of its infected neighbors in the other network with probability *β*_2_. For example, if a susceptible node has *m* infected neighbors in the same network and *n* infected neighbors in the other network, the probability that it will become infected is 1-(1-*β*_1_)^*m*^(1-*β*_2_)^*n*^. Meanwhile, each infected nodes can be cured and return to the susceptible state with probability δ. The effective spreading rates are defined as *λ*_1_ = *β*_1_/*δ* and *λ*_2_ = *β*_2_/*δ*. Without loss of generality, we set *δ* = 1 and *λ*_1_ = *λ*_2_ = *λ*.

## Results

The basic parameters that characterize epidemic spreading are the epidemic threshold λ_c_ and the infection density *ρ*. If the effective infection rate *λ* is above the threshold, *λ*≥ *λ*_*c*_, then the epidemic will spread and the system will ultimately have a non-zero steady-state infection density (*ρ* > 0). By contrast, when *λ< λ*_*c*_, the epidemic will die out (*ρ* = 0).

We explore the effect of the rewiring probability of the interconnected networks on the epidemic spreading. We perform simulations to obtain the infection density *ρ* in the steady state as a function of the effective infection rate *λ* for various rewiring probabilities. Initially, 10% of the nodes in network A are randomly chosen to be infected. The initial infection of 10% nodes are only in layer A, and nodes of layer B can get infected due to the interconnections with layer A in the following steps.

**Fig 2:**
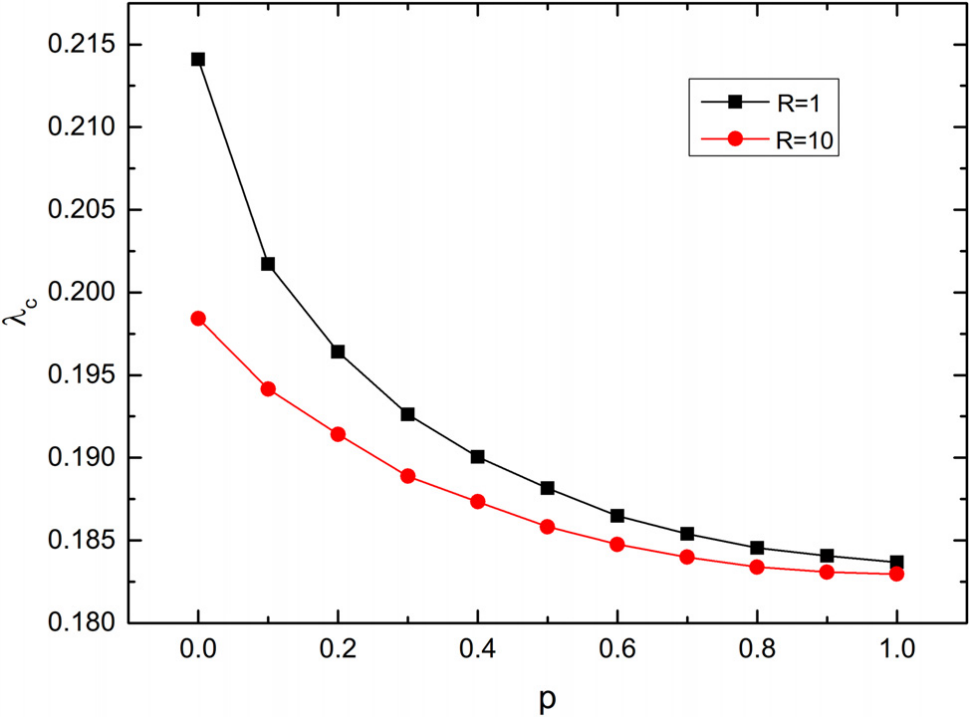
Epidemic threshold *λ*_*c*_ as a function of the rewiring probability for different spatial constraints *R* on the interconnection links. For a given rewiring probability *p* and a given spatial constraint *R*, we gradually increase the infection rate *λ* and find the corresponding infection density in the steady state for each infection rate. We consider *λ*_*c*_ as the first *λ* value corresponding to a non-zero infection density in the steady state. Each component network has a small-world topology with a rewiring probability *p*. The density of the interconnection links is *q* = 1. Initially, 10% of the nodes in network A are randomly chosen to be infected. The network size is *N* = 10000. The results have been averaged over 100 realizations.

To study the effect of the small-world network features on the epidemic threshold *λ*_*c*_, we plot the epidemic threshold *λ*_*c*_ as a function of the rewiring probability *p*. As shown in Fig. 2, for a fixed *R*, the epidemic threshold decreases as *p* increases. This is because, as *p* increases, more links in the original lattices A and B are rewired, reducing the average distance of both networks, which enhances the spreading of epidemic. And as *R* increases, while the number of interconnections stays the same, the interconnections can bridge distinct locations, which facilitates the epidemics spreading. When *p* is small, the impact of the spatial length of the interconnections on the epidemic threshold is significant. When *p* is large, however, the epidemic threshold is barely influenced by this spatial length. Both high rewiring probability *p* and large spatial length *R* contribute to the heterogeneity of the connections formed in the interconnected networks, which is beneficial to the spreading of epidemics. This explains why a high rewiring probability could actually diminish the effect of spatial constraints on the topology of the interconnected networks.

**Fig 3:**
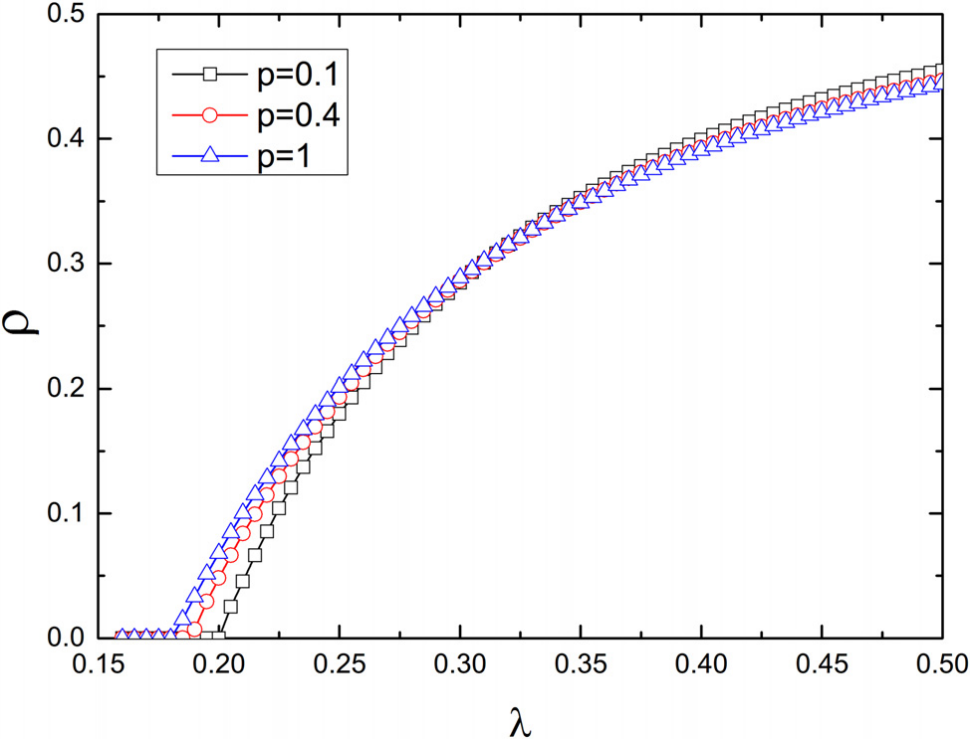
Density *ρ* of infected nodes as a function of the infection rate *λ* for various rewiring probabilities. *ρ* is the average of the infection density *ρ*_*A*_ and *ρ*_*B*_. The density of the interconnection links is *q* = 1, and the spatial length constraint is *R* = 1. Initially, 10% of the nodes in network A are randomly chosen to be infected. The network size is *N* = 10000. The results have been averaged over 100 realizations.

As shown in Fig. 3, interconnected networks with a larger rewiring probability *p* have a smaller epidemic threshold *λ*_*c*_, confirming the results presented in Fig. 2. When the infection rate *λ* (greater than *λ*_*c*_) is small, the average infection density *ρ* of interconnected networks with a higher rewiring probability is larger than that of interconnected networks with a small rewiring probability. As the infection rate is further increased, however, the infection density of the interconnected networks is hardly influenced by the rewiring probability.

**Fig 4:**
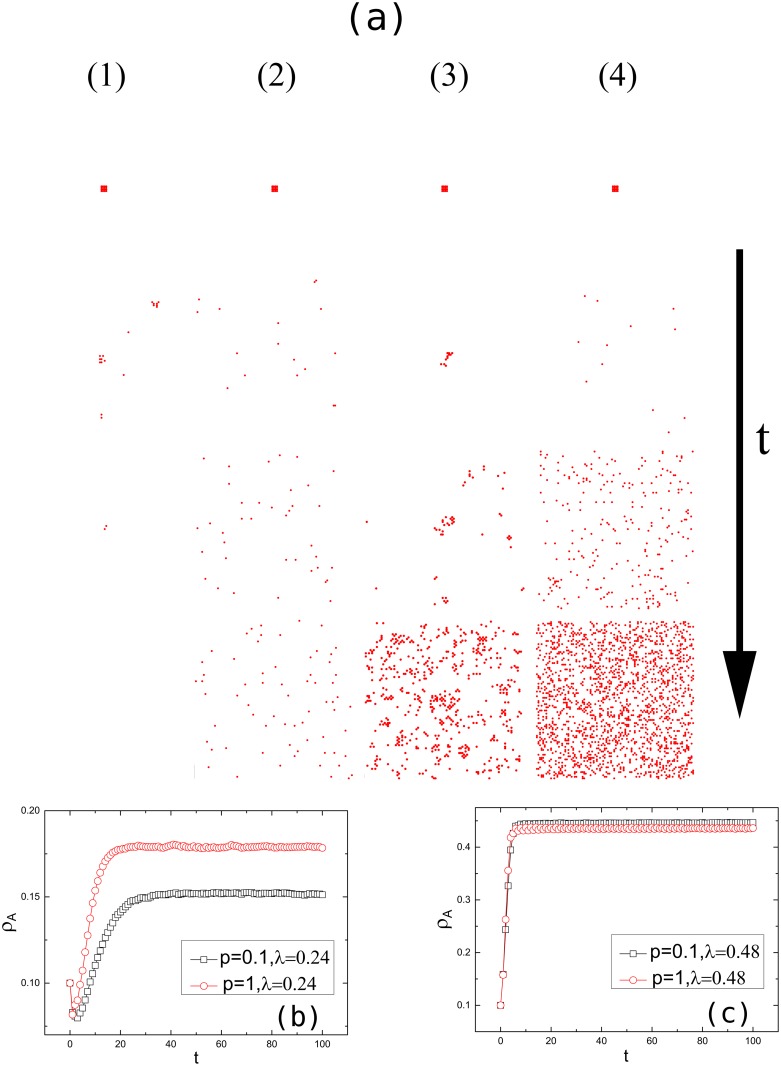
Epidemic spreading process in interconnected small-world networks. (a) illustrates the epidemic spreading pattern in network A. Initially, 16 nodes in the center of network A are infected. The rewiring probabilities are *p* = 0.1 ((1), (3)) and *p* = 1 ((2), (4)), and the infection rates are *λ* = 0.19 ((1), (2)) and *λ* = 0.24 ((3), (4)). (b) and (c) present the infection density *ρ*_*A*_ in network A as a function of time for two interconnected networks. Initially, 10% of the nodes in network A are randomly chosen to be infected. The infection rates are *λ* = 0.24 (b) and *λ* = 0.48 (c). The density of interconnection links is *q* = 1. The spatial length is *R* = 1. The network size is *N* = 10000. The results have been averaged over 100 realizations.

To discover the different spreading patterns of epidemics, we investigate the epidemic spreading over space and time, considering various rewiring probabilities and infection rates. Initially, 16 nodes in the center of the network A are infected. From (1) and (2) of Fig. 4(a), it is evident that when the infection rate is *λ* = 0.19 and the rewiring probability is *p* = 0.1, the disease spreads only within local clusters and ultimately dies out; by contrast, when the infection rate *λ* = 0.19 is the same but the rewiring probability is *p* = 1, the disease spreads throughout the network and persists with a non-zero infection fraction. We further explore the case when the infection rate is *λ* = 0.24. As shown in (3) and (4) of Fig. 4(a), the infected nodes initially infect predominantly their local neighbors when *p* is small; when *p* is large, however, nodes far away from the initially infected nodes may get immediately infected.

We further study the time evolution of the infection density *ρ* for various rewiring probabilities and infection rates. As shown in Fig. 4(b) and also observed in (3) and (4) of Fig. 4(a), when the infection rate is relatively low e.g. *λ* = 0.24, above but close to the epidemic threshold, a higher rewiring probability leads to a faster spread and a higher steady-state infection density. When the infection rate is sufficiently high e.g. *λ* = 0.48, as shown in Fig. 4(c), the disease propagates rapidly in both networks and the rewiring probability has minor influence on the infection density.

**Fig 5:**
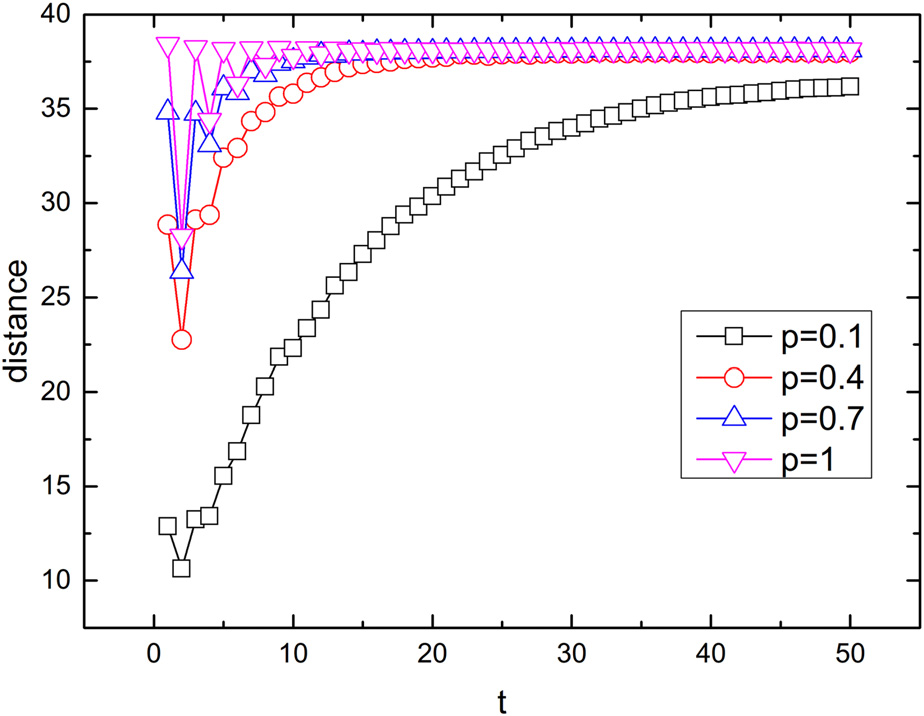
Time evolution of the average distance of infected nodes from the center of the lattice in network A. The infection rate is *λ* = 0.24. Initially, 16 nodes in the center of network A are infected. The density of interconnection links is *q* = 1. The spatial length is *R* = 1. The network size is *N* = 10000. The results have been averaged over 100 realizations.

To understand how fast an epidemic spreads spatially, we examine the average distance of the infected nodes in network A from the center of the lattice as a function of time in Fig. 5. When *p* is small, the average distance gradually increases to the maximum distance at a low velocity. As *p* increases, epidemics can spread spatially all over the network with less time due to the addition of shortcuts. This finding is consistent with the results presented in Fig. 4(a).

## Conclusions

In summary, we studied the spread of epidemics in interconnected small-world networks with spatial constraints. We found that the rewiring probability of the small-world networks strongly affects the epidemic spreading behavior. We demonstrated that the epidemic threshold decreases as the rewiring probability increases. When the infection rate is low, the steady-state infection density varies with the rewiring probability. However, when the infection rate is sufficiently high, the infection density does not differ considerably for different rewiring probabilities.

While previous studies have focused on the epidemics spreading on single networks, recent work on viral spreading in interconnected and multilayer networks reveals new phenomena that cannot be captured in a single network. We deem the development of immunization and vaccination strategies based on the realistic interconnected and multilayer networks as the promising and challenging future work [29,30].
